# How Did the Built Environment Affect Urban Vibrancy? A Big Data Approach to Post-Disaster Revitalization Assessment

**DOI:** 10.3390/ijerph191912178

**Published:** 2022-09-26

**Authors:** Hongyu Gong, Xiaozihan Wang, Zihao Wang, Ziyi Liu, Qiushan Li, Yunhan Zhang

**Affiliations:** 1 School of Architecture and Environment, Sichuan University, Chengdu 610065, China; 2 Wuyuzhang Honors College, Sichuan University, Chengdu 610065, China; 3 Institute for Disaster Management and Reconstruction, Sichuan University, Chengdu 610065, China

**Keywords:** post-disaster reconstruction, multi-source data, geovisual analytics, geospatial model, sustainable development

## Abstract

Quantitative assessment of urban vibrancy is crucial to understanding urban development and promoting sustainability, especially for rapidly developing areas and regions that have experienced post-disaster reconstruction. Taking Dujiangyan City, the hardest-hit area of the earthquake, as an example, this paper quantifies the urban economic, social, and cultural vibrancy after reconstruction by the use of multi-source data, and conducts a geographic visualization analysis. The purpose is to establish an evaluation framework for the relationship between the urban built environment elements and vibrancy in different dimensions, to evaluate the benefits of post-disaster restoration and reconstruction. The results show that the urban vibrancy reflected by classified big data can not be completely matched due to the difference in the data generation and collection process. The Criteria Importance Though Inter-criteria Correlation and entropy (CRITIC-entropy) method is used to construct a comprehensive model is a better representation of the urban vibrancy spatial characteristics. On a global scale, comprehensive vibrancy demonstrates high continuity and a bi-center structure. In the old town, the distribution of various urban vibrancies show diffusion characteristics, while those in the new district demonstrated a high degree of aggregation, and the comprehensive vibrancy is less sensitive to land-use mixture and more sensitive to residential land.

## 1. Introduction

Urban vibrancy is an important concept for evaluating the level of urban development, which reflects the attractiveness of an area to both people and activities [1]. Numerous studies have investigated the urban vibrancy concept and contended that it stems from human activity and spatial diversity [2]. According to Gehl, human spontaneous and social activities are key to the vibrancy of public spaces [3]. According to Montgomery, vibrant public spaces often nurture vibrant urban activities [4]. In general, urban vibrancy is the spatial behavior that occurs in urban space due to productive, living, and spiritual needs. It also typically results from interactions between people and urban spaces [5], and is a visual representation of urban development. Therefore, understanding the intrinsic mechanisms of urban vibrancy, namely the interrelationship between urban vibrancy and its influencing factors, can help stimulate urban vibrancy and thus promote scientific and sustainable development in the planning field [6].

The depiction of urban vibrancy and the best explanatory variables to use are trending research areas. The use of big data to evaluate urban vibrancy has become mainstream urban research owing to the proliferation of large datasets [5,7,8]. Examples include the use of cell phone signaling data [9], social media data [6,8,10], point-of-interest data [10,11], Baidu heat map data [12,13,14], and Nighttime light imagery [15]. Such data are always acquired by massive small portable devices, thereby having strong timeliness, and able to record the activity areas and type of activities of a large number of individuals in different time periods [16,17]. Meanwhile, they also contain a whole bunch of urban interactive information, which is conducive to depicting the spatial-temporal pattern of urban vibrancy, thus beneficial to those scholars and policy-makers who need to have a sound grasp of the operation of cities to make better urban studies and policy decisions [18]. Researchers have mostly used a single data source to characterize urban vibrancy [8,19,20]; however, as a giant complex system, the vibrancy of cities are usually affected by many factors, such as society, economy, culture, and so on [21]. Therefore, it is necessary to select multiple big datasets to characterize the different dynamics of cities and integrate them into comprehensive dynamics [11,22]. In this research, urban vibrancy is divided into three dimensions according to the post-disaster reconstruction master plan: social, economic, and cultural vibrancy. Urban social vibrancy is defined as the intensity of the flow of people who participate in various activities in the city [23], urban economic vibrancy refers to the intensity of urban residents engaged in economic activities, such as production and consumption, which, to a certain extent, represents the potential of urban development, and is also used as an index to measure urban safety [23]. Urban cultural vibrancy refers to the creation, storage, and dissemination of the city’s spiritual culture, which is the vibrancy produced by the residents’ pursuit of higher-level spiritual needs [11].

Most studies have focused on cities or regions in a steady-state of development, and few have examined particular stages of urban development [4,20,24]. However, urban vibrancy evaluation results may not be consistent for cities with different development stages and construction situations [25]. Dujiangyan City is one of the Wenchuan earthquake’s hardest-hit areas. Its recovery and reconstruction plan can be divided into four phases: the completion of movable housing within three months; completion of permanent resettlement within three years; promotion of comprehensive socioeconomic development in the affected area within five years; and socioeconomic development above pre-quake level within eight years. Dujiangyan is currently in the final stage of reconstruction, and it is crucial to evaluate this stage from the perspective of urban vibrancy combined with big data to systematically understand the development of the city following the disaster. This study uses Dujiangyan City as the study area, constructs a comprehensive urban vibrancy evaluation model using multi-source big data, analyzes the geospatial distribution characteristics of different metrics of urban vibrancy, and quantitatively evaluates the global and local impact characteristics of built environment elements on different urban vibrancy in Dujiangyan using ordinary least squares (OLS) and geographically weighted regression (GWR) models.

The study attempts to contribute to urban vibrancy assessment in several ways: (1) expand the use of Baidu heat maps in urban planning research by extracting heatmaps to characterize crowd activities and discuss social vibrancy. (2) Construct a comprehensive urban vibrancy model that can accurately describe the spatial pattern of urban vitality in post-disaster reconstruction. (3) Further analyze the influence mechanism of built environment elements on urban vitality in different dimensions, and put forward practical suggestions for the rational planning of post-disaster reconstruction cities.

## 2. Materials and Methods

### 2.1. Study Area

**Figure 1:**
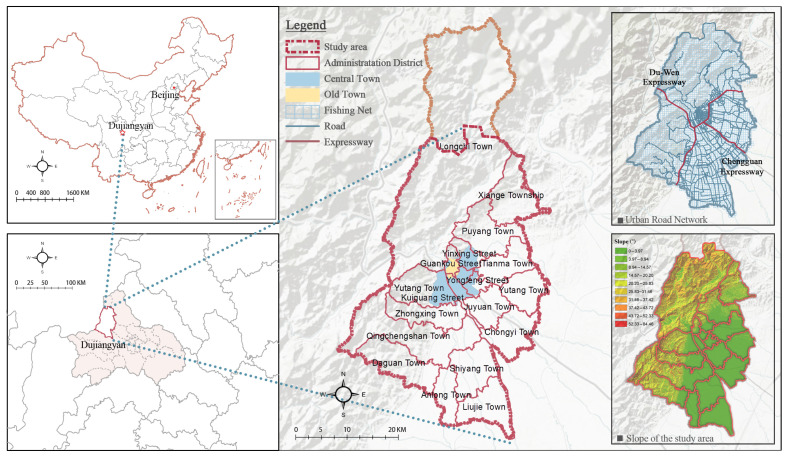
Study area: Dujiangyan, China.

Dujiangyan, a city most severely affected by the 2008 Wenchuan earthquake, is located on the northwest edge of the Chengdu Plain in central Sichuan Province [26,27]. Dujiangyan has six street offices: Happiness, Gunkou, Kuiguangta, Yinxing, Puyang, and Yutang; five towns: Juyuan, Tianma, Longchi, Qingchengshan, and Shiyang; and one economic development zone (Figure 1). A total of 625,100 people were affected by the 2008 Wenchuan earthquake, resulting in a direct economic loss of 53.676 billion yuan. Two years after the earthquake, Dujiangyan completed the reconstruction of urban housing at its original location [26]. Three years after the earthquake, public livelihood projects completed 86.3% of the target tasks of restoration and reconstruction. It is currently in the final stages of recovery, reconstruction, upgrading, and development, with a gross domestic product (GDP) of 44.17 billion yuan in 2021 already, a growth of 29.55 billion yuan compared with the pre-earthquake period (2007).

To more accurately quantify and evaluate the urban vitality of Dujiangyan post-disaster development. In the case of fully considering the distribution of population and social resources, the natural ecological reserve in the northern part of the city is not in the scope of this paper, and the emphasis was placed on the plain area in the southeast of the city. The basic analysis unit of this study was a 500 m × 500 m grid. As shown in Figure 1, the study area was divided into 4188 spatial grids.

### 2.2. Data Sources

The datasets used in this study were the Baidu Heat Map (BHM) dataset, Nighttime light (NTL) imagery dataset, point-of-interest (POI) dataset, and built environment dataset (including road networks, public transportation stations, and land-use data).

The BHM dataset is based on user-generated location data. The BHM dataset used in this study was downloaded from Baidu Map API (https://map.baidu.com/ (accessed on 20 May 2022 and 22 May 2022)) every two hours from 6:00 to 24:00 on Friday, 20 May 2022, and Sunday, 22 May 2022. The data have a resolution of 50 m × 50 m. The nighttime light remote sensing data are from the Suomi National Polar-orbiting Partnership (Suomi-NPP), which carries the visible infrared imaging radiometer suit (VIIRS) with a spatial resolution of 417 m × 417 m. The NPP/VIIRS Nighttime light remote sensing month-by-month data from January to December 2021 were selected. The data were sourced from the National Centers for Environmental Information Earth Observation Group VIIRS Nighttime light (https://eogdata.mines.edu/products/vnl/ (accessed on 8 May 2022)), an affiliation of the National Oceanic and Atmospheric Administration. POI data were obtained from Baidu Maps (https://map.baidu.com/) (accessed on 1 August 2021), which consists of 184 sub-categories in 22 major categories, totaling 38,600 points, were reclassified into 12 categories based on related studies and urban land classification and construction land planning standards (GB50137-2011) (refer to Table A1 in Appendix A) [25], and then extracted POIs of science, education, culture (1095) and public transportation stations (2187). Science, education, and culture POI include art museums, science and technology museums, museums, planetariums, libraries, cultural palaces, theaters, concert halls, exhibition halls, convention centers, scientific research institutions, and schools. Road network data were obtained from the Chengdu Road Traffic Operation Index System (http://www.cdtdri.com/ (accessed on 15 May 2022)). Land-use data were obtained from the “Dujiangyan Land-use Master Plan (2006–2020)’’.

In this paper, research data were mainly collected during the COVID-19 pandemic. Due to the strict social control measures taken by the Chinese government after the year 2020, coupled with the characteristic of Dujiangyan City as a tourist city, it will affect the vibrancy of the city to a certain extent. In order to carry out the research in the context of a relatively objective assessment, by consulting the 2017–2021 “Statistical Bulletin of Dujiangyan City’s National Economic and Social Development”, it is found that the impact of the epidemic on tourism in Dujiangyan was not significant. Moreover, the 2021 Dujiangyan epidemic risk control policy includes only one community, involving 600 people, and the influence on the overall urban vibrancy trend is negligible.

### 2.3. Methods

**Figure 2:**
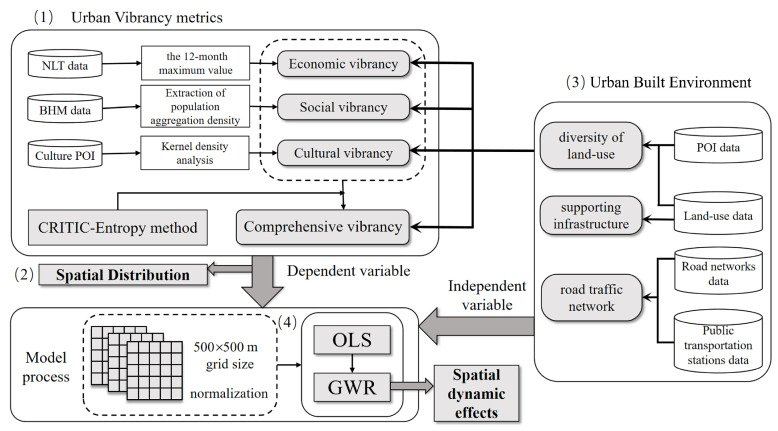
Research framework.

This study constructed an evaluation system for urban vibrancy (Figure 2). It includes four steps: (1) quantifying the economic vibrancy, social vibrancy, and cultural vibrancy of cities using three types of big data: NTL, BHM, and POI, and using the CRITIC-entropy method to construct a comprehensive urban vibrancy model; (2) visualizing the four types of vibrancy and comparing their spatial distributions; (3) selecting possible influence factors to construct a built environment index system; and (4) applying the OLS and GWR models to exploring the correlation between built environment factors and urban vibrancy.

#### 2.3.1. Urban Vibrancy Indicators

(1) City Social Vibrancy Index

The Baidu heat map is location-data generated by users of Baidu products using location-based services, which can reflect the spatio-temporal activities of people. Due to its accuracy in spatial and temporal scales, it has been widely used in studies closely related to human activities [12,13,14]. Urban social vibrancy are closely related to residents’ activities and trips, and can be quantified by population density [28,29]. Zhang proposed a method for determining the population aggregation density using the pixel value of the BHM [30]. Based on his study, this paper proposes a method for measuring social vibrancy using the pixel values of BHM.

First, this study characterizes the pixel value of the *h*th grid on day *d* in terms of the maximum average pixel value of the *h*th gird in each period of day *d* ($P_{h,d}$) (*d* = 1 for week days, *d* = 2 for rest days), as follows:

(1) $$P_{h,d}=\max_{t}\frac{\sum_{i=1}^{m}c_{h,d,t,i}}{m}$$

where m is the total number of raster for storing pixels contained in each fishnet grid, and $c_{h,d,t,i}$ is the pixel value stored in the *i*th raster in the *h*th fishnet grid at time *t* on day *d*.

The pixel value of BHM showed a linear relationship with the population aggregation density when it was less than 151 [30]. The *h*th grid pixel values for both weekends and weekdays were calculated to be less than 151, at which point the grid pixel values and the cell population aggregation density showed a linear relationship and their values were equal following the min–max normalization. Therefore, normalized pixel values can be used to represent the aggregation of people and thus quantify the social vibrancy. The resulting social vibrancy index for a *i*th grid on weekdays and the social vibrancy index for the *i*th grid on weekends are $USV1_{h}$ and $USV2_{h}$, respectively.

Considering that the distribution of the population on weekends and weekdays shows special differences [12,13,28], according to the proportion of weekdays and weekends in a week, we weighted the average of $USV1_{h}$ and $USV2_{h}$, and then normalized the average to obtain the social vibrancy index of each fishnet grid ($USV_{h}$).

(2) $$USV_{h}=\frac{\left(\frac{5USV1_{h}+2USV2h}{7}\right)-\min_{h}\left(\frac{5USV1_{h}+2USV2}{7}\right)}{\max_{h}\left(\frac{5USV1_{h}+2USV2}{h}\right)-\min_{h}\left(\frac{5USV1_{h}+2USV2_{h}}{7}\right)}$$

(2) City Cultural Vibrancy Index

POI data are a widely used urban big data that can accurately reveal urban functions and their spatial location information for urban function inference [31]. It is highly flexible in terms of research scale [10]. This research quantified urban cultural vibrancy with science, education and culture POI, specifically using kernel density analysis. This method was used to obtain the density values of point and line element neighbors in geographical space and then simulate them in a spatially continuous manner. It is now widely used to study the spatial distribution characteristics of elements, such as monocentric or polycentric distributions [32]. This study uses the density value of cultural facilities POI in each fishing grid’s midpoint to characterize urban cultural vibrancy. The formula is expressed as follows.

(3) $$\lambda_{h}=\sum_{i=1}^{n}\frac{1}{\pi r^{2}}k\left(\frac{d_{ih}}{r}\right)$$

where *h* denotes the midpoint of *h*th grid, $\lambda_{h}$ denotes the kernel density at *h*, *r* denotes the search radius, $d_{ih}$ represents the distance from *i* to *h* (Euclidean distance was used in this study), and *k* denotes the kernel function (Gaussian function was used in this study).

The min–max normalization was then used to obtain the cultural vibrancy index of each grid ($UCV_{h}$).

(4) $$UCV_{h}=\frac{\lambda_{h}-\min_{h}\left(\lambda_{h}\right)}{\max_{h}\left(\lambda_{h}\right)-\min_{h}\left(\lambda_{h}\right)}$$

(3) City Economic Vibrancy Index

Compared with artificial statistics, nighttime light data can directly capture the spatial distribution of artificial radiation in human settlements with less error [33]. It can be used to visualize the real-time state of economic activity and intensity of human activity [34], and is commonly used to assess urban economic activity [35]. This study uses nighttime light data to quantify urban economic vibrancy by preprocessing the month-by-month lighting data using operations to remove lighting outliers and derive the average pixel value within each grid ($P_{h,t}$).

(5) $$P_{h,t}=\frac{\sum_{i=1}^{m}c_{h,t,i}}{m}$$

where *m* is the total number of raster with pixels stored in this fishnet grid, and $c_{h,t,i}$ denotes the pixel value stored in the *i*th raster contained in the *h*th fishing grid in month *t*.

Owing to the relatively low accuracy of nighttime atmospheric correction and cloud detection, this study further uses the maximum grid-averaged pixel value of 12 months as a characterization of urban economic vibrancy, and derives the grid’s economic vibrancy index ($UEV_{h}$) by min–max normalization.

(6) $$UEV_{h}=\frac{\max_{t}P_{h,t}-\min_{h}\left(\max_{t}P_{h,t}\right)}{\max_{h}\left(\max_{t}P_{h,t}\right)-\min_{h}\left(\max_{t}P_{h,t}\right)}$$

(4) Comprehensive City Vibrancy Assessment

The description of the overall urban vibrancy level by a single datum is somewhat biased [36]; therefore, it is essential to compare and analyze urban vibrancy characterized by different data sources to build a comprehensive urban vibrancy for urban vibrancy research [11]. Considering that various types of vibrancy play different roles in comprehensive urban vibrancy [36], subjective choices may lead to inaccurate representations of the spatial characteristics of comprehensive vibrancy. In this study, a combined evaluation weighting method based on the entropy method and the CRITIC method was used to measure the impact of each vibrancy on the comprehensive urban vibrancy. The CRITIC method is an objective weighting method, which compares the strengths of indicators and conflicting indicators [37]. It integrates data fluctuations and correlations between indicators, but ignores the amount of information those indicators carry. Conversely, the entropy weighting method determines the indicator weights by considering the information volume of the indicators according to the dispersion degree among the indicators [38]. The CRITIC and entropy weight methods can be used together to fully consider the similarities and differences between various vibrancy [39]. The calculation processes are as follows.

The entropy weight method was used first to calculate the weight of each vibrancy index ($w1_{j}$).

a. Calculation of information entropy

(7) $$E_{j}=-\frac{1}{\ln(m)}\sum_{i=1}^{m}\left(\frac{x_{hj}}{\sum_{i=1}^{m}x_{hj}}\right)\ln\left(\frac{x_{hj}}{\sum_{i=1}^{m}x_{hj}}\right)$$

Equation (7) presents a method to calculate the information entropy of *j*-vibrancy ($E_{j}$), where $x_{hj}$ denotes the j-vibrancy index calculated from *h*th grid (*j* = 1 for economic vibrancy, *j* = 2 for social vibrancy, and *j* = 3 for cultural vibrancy), and *m* denotes the total number of fishnet grids. The smaller $E_{j}$ is, the higher the degree of variation of its indicator value, the greater the amount of information provided, and the stronger the influence of the indicator on the comprehensive vibrancy.

b. Calculation of weights

(8) $$w1_{j}=\frac{1-E_{j}}{k-\sum_{j=1}^{k}E_{j}}$$

Equation (8) essentially states the *j*-vibrancy’s weight calculated by the entropy method. The higher $w1_{j}$ is, the greater the *j*-vibrancy contribute to the comprehensive vibrancy.

Next, the CRITIC method was then applied to calculate the weights of each vibrancy index ($w2_{j}$).

a. Calculation of vibrance contractility

(9) $$\sigma_{j}=\sqrt{\frac{\sum_{h=1}^{m}\left(x_{hj}-{\bar{x}}_{j}\right)}{m-1}}$$

Equation (9) essentially measures the contractility of *j*-vibrancy ($\sigma_{j}$), where ${\bar{x}}_{j}$ denotes the mean value of *j*-vibrancy of all cells. The higher $sigma_{j}$ is, the greater the difference in its values is, the more special information may be reflected, the more strongly *j*-vibrancy influences the comprehensive vibrancy, and the more weight of *j*-vibrancy should be.

b. Calculation of vibrancy paradoxicality

(10) $$f_{j}=\sum_{i=1}^{m}\left(1-r_{ij}\right)$$

Equation (10) essentially measures the paradoxicality of *j*-vibrancy ($f_{j}$), where $r_{i,j}$ denotes the correlation coefficient between the *i*-vibrancy and *j*-vibrancy. The Pearson’s correlation coefficient was used in this study. The weight of *j*-vibrancy should increase because the larger $f_{j}$ is, the less the correlation with the rest of the vibrancy is, reflecting more special information.

c. Calculation of the weights of different vibrancy

(11) $$w2_{j}=\frac{\sigma_{j}f_{j}}{\sum_{j=1}^{k}\sigma_{j}f_{j}}$$

Equation (11) essentially states the weight of *j*-vibrancy ($w2_{j}$) calculated by the CRITIC method. The higher $w2_{j}$ is, the greater the *j*-vibrancy contributes to the comprehensive vibrancy.

Finally, the combination weight of *j*-vibrancy ($w_{j}$) is calculated.

(12) $$w_{j}=\beta w1_{j}+\left(1-\beta \right)w2_{j}$$

In this study, the significance of the two assignment methods is assumed to be equal; therefore, β is set at 0.5.

By the using of the above calculation method, the urban social vibrancy index, the urban cultural vibrancy index and the urban economic vitality index are integrated, and then the results are normalized to construct a comprehensive urban vibrancy ($UV_{h}$).

(13) $$UV_{h}=\frac{\left(w1UEV_{h}+w2USV_{h}+w3UCV_{h}\right)-\min_{h}\left(w1UEV_{h}+w2USV_{h}+w3UCV_{h}\right)}{\max_{h}\left(w1UEV_{h}+w2USV_{h}+w3UCV_{h}\right)-\min_{h}\left(w1UEV_{h}+w2USV_{h}+w3UCV_{h}\right)}$$

#### 2.3.2. Built Environment Elements

Based on the characteristics of the data and the social context of urban post-disaster reconstruction [6,25,38], the built environment elements selected for this study suggest that the determinants of urban vibrancy lies in three areas: the diversity of land-use (land-use mixture, proportion of residential land), supporting infrastructure (proportion of commercial land, proportion of public amenity land), and road traffic networks (closeness, betweenness, distance to bus stops) (refer to Table A2 in Appendix A).

*Land-Use Mixture:* The Simpson index reflects the richness of POI and the relative abundance of different types of POI. The diversity of the land-use increases as the index value increases. In this study, the Simpson index was selected to calculate the mixed use of 12 POI types and to explore how spatial resource allocation affects urban vibrancy [9].

*Land-Use Proportion*: Urban vibrancy evaluation systems have been studied to include the proportion of commercial and facility land-use to capture the spatial distribution of urban land-use [20,40]. In this study, the proportion of residential land was one of the indicators of land-use diversity, while the proportion of commercial and public amenity land were the main indicators of supporting infrastructure. The proportions of residential, commercial, and public amenity land areas in each unit were calculated separately.

*Road Closeness and Betweenness*: Spatial syntax is a model that is used to investigate the interaction between urban form and function and is mediated by the movement of people and vehicles, establishing spatial and social associations [38]. The spatial design network analysis (sDNA) model, developed by the School of Geography and Planning, Cardiff University, UK, in 2013, aims to improve the modeling and analysis capabilities of spatial syntax for large-scale cities and regions. In this study, the closeness and betweenness were chosen as the sDNA model parameters.

Closeness represents a measure of how easy it is for a road to reach other roads within the search radius. Road with high closeness usually have high topological integration and, thus, exhibit high accessibility and centrality [40]. The calculation formula is as follows:

(14) $$NQPD\left(x\right)=\sum_{y\in R_{x}}\frac{{\left(W\left(y\right)P\left(y\right)\right)}^{nqpdn}}{d_{M}{\left(x,y\right)}^{nqpdd}}$$

where NQPD(x) represents the closeness at point *x*, $R_{x}$ denotes the search radius at point *x*, *W(y)* denotes the weight of chain *y*, *P(y)* denotes the weight of node y within the search radius $R_{x}$, besides, *nqpdn* and *nqpdd* are both set at one. This study is a continuous analysis; therefore, *P(y)*∈[0,1]; $d_{M}\left(x,y\right)$ represents the shortest topological distance from node *x* to node *y* (Equation (14)).

Betweenness is often used to measure the probability of a road segment being passed by traffic flow and to respond to the potential of the road segment, with higher betweenness representing a higher probability of the road network being passed [41]. The calculation method is as follows:

(15) $$OD\left(y,x,z\right)=\left\{\begin{array}{cc} 1, & \mathrm{If}\;x\;\mathrm{lies}\;\mathrm{on}\;\mathrm{the}\;\mathrm{shortest}\;\mathrm{path}\;\mathrm{from}\;y\;\mathrm{to}\;z\\ \frac{1}{2}, & \mathrm{if}\;x=y\neq z\\ \frac{1}{2}, & \mathrm{if}\;x=z\neq y\\ \frac{1}{3}, & \mathrm{if}\;x=y=z\\ 0, & \mathrm{Other}\;\mathrm{cases} \end{array}\right.$$

(16) $$\mathrm{TPBt}\left(x\right)=\sum_{y\in N}\sum_{N_{t}\in R_{y}}OD\left(x,y,z\right)\frac{W(x)P(x)}{W(y)}$$

where TPBt(*x*) denotes the betweenness at *x*, *OD(x,y,z)* denotes the shortest topological distance between *y* and *z* through node *x* in the search radius, and *W(y)* denotes the total weight of *y* in the search radius.

**Figure 3:**
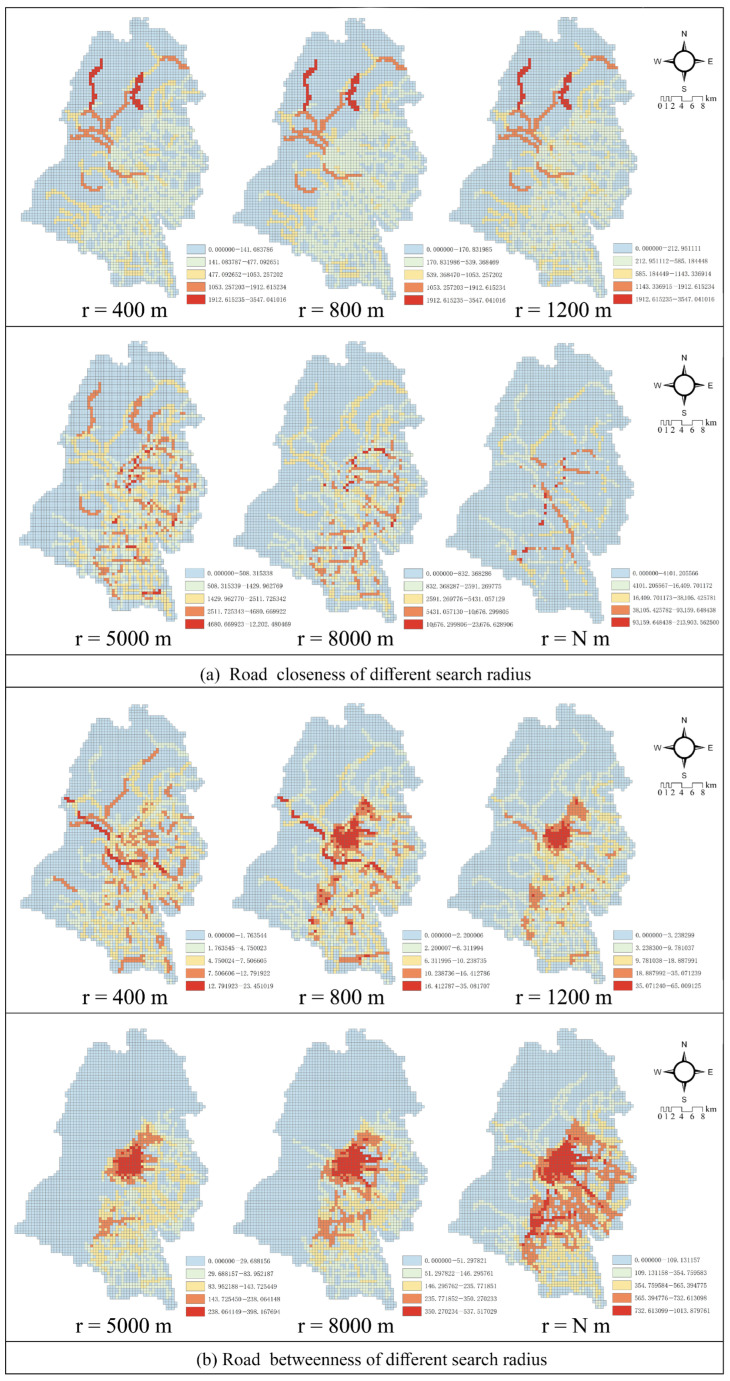
Road closeness (**a**) and betweenness (**b**) of different search radius. The search radius equal to 400 m, 800 m, 1200 m, 5000 m, 8000 m, and N m.

Yang’s study (2007) on the spatial networks of several urban clusters indicated that there are differences between the spatial network structures obtained from analysis at different scales [42]. In this study, based on the relationship between the residents’ daily life travel and the actual distance, the search radius of 400 m, 800 m, 1200 m, 5000 m, 8000 m, and N m (N means infinity) were set for the spatial form of the research object (Figure 3).

To eliminate the influence of magnitudes among indicators and, thus, better explain urban vibrancy, this study performs min–max normalization in the above results to obtain the land-use mixture index, residential land index, commercial land index, public amenity land index, distance index, road closeness index, and road betweenness index. As shown in Table 1, the elements of the built environment and their index are as follows (Table 1).

## 3. Results

### 3.1. Spatial Distribution of Urban Vibrancy

**Figure 4:**
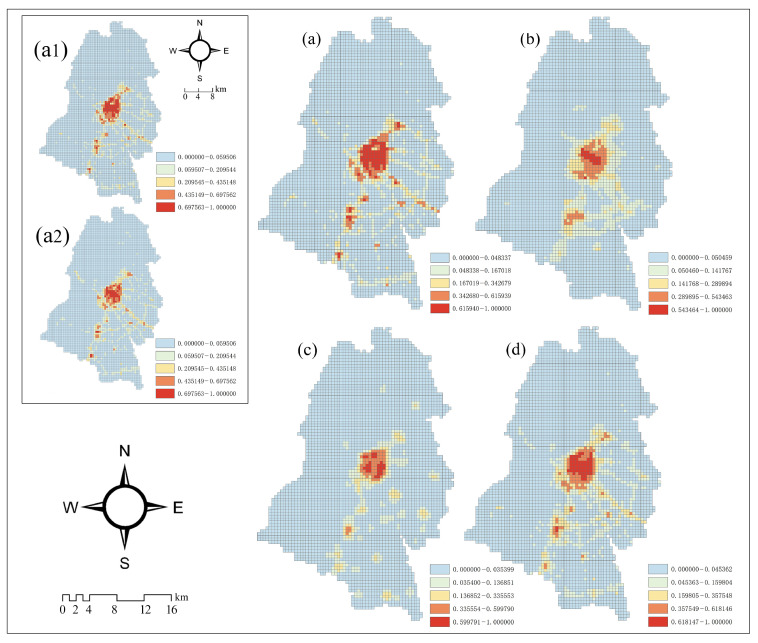
Spatial patterns of urban vibrancy in Dujiangyan. (**a**) Urban Social Vibrancy (USV). (**a1**) Urban Social Vibrancy on weekdays (USV1). (**a2**) Urban Social Vibrancy on weekends (USV2). (**b**) Urban Economic Vibrancy (UEV). (**c**) Urban Cultural Vibrancy (UCV). (**d**) Urban Comprehensive Vibrancy (UV).

Figure 4 shows different spatial patterns of urban vibrancy in Dujiangyan City. The distribution of USV, UEV, and UCV appears similar. They are prominent in the central city and Qingchengshan New District, showing a “double-center’’ character, and forming “sub-centers’’ in other town centers. From Figure 1, the distribution of urban vibrancy coincides with the trend of the main roads in the city, and its value shows a gradient decrease from the urban core to the suburban areas. In addition, the city’s southeast is where the most of the high value urban vibrancy is concentrated, whereas the western and northern mountainous regions of the city are where the majority of the low value regions are found.

There are differences in the distribution of local vibrancy according to USV, UEV, and UCV. For example, the aggregation of the USV and UCV can be observed at the southern edge of the city, whereas the UEV cannot. Moreover, the aggregation of UCV and USV could be observed at the southern end of the city, while UEV could not be observed. There are also differences in their spatial continuity, with UCV having poorer continuity and more dispersed spatial distribution, while UEV and USV show better spatial continuity. Comparing the spatial distribution of UV and these three, it is found that UV is consistent with them globally and can better express their respective important features locally.

The USV can be discussed from two perspectives: weekdays and weekends. According to Figure 4(a1,a2), the distribution of high-value areas in USV1 (Urban social vibrancy on weekdays) is more concentrated in the central city than it is in USV2 (Urban social vibrancy on weekends). Moreover, the USV gather on the main roads of the city (particularly the Chengguan Expressway), as well as at scenic spots (Figure 1). Three of the four high-value areas of UCV in the central city were located in the old town with rich cultural and tourism resources (Figure 4c).

### 3.2. Results of the Global Regression Model

The OLS was used in this study to examine the overall relationship between the different vibrant and built environment characteristics (Table 2). The selected built environment variables were significantly correlated with urban social vibrancy (model1), urban cultural vibrancy (model2), urban economic vibrancy (model3), and comprehensive urban vibrancy (model4) at a confidence level of 0.001 with the highest degree of explanation (77.6%) for comprehensive urban vibrancy.

According to Table 2, LMI, RLI, CLI, PALI, RCI, and were all positively related to the four types of vibrancy. The RCI had the highest correlation coefficient among the four regression models. This means that after restoration and reconstruction, the area with high closeness in Dujiangyan had a high level of urban vibrancy. Vibrancy and DI and RBI were negatively correlated, and the correlation is RBI > DI. This suggests that a high degree of betweenness does not necessarily play a role in promoting urban vibrancy; instead, it may lead to a lower level of vibrancy.

### 3.3. Results of Geographically Weighted Regression

Because OLS can only respond to the influence of various built environment elements on urban vibrancy at the global scale, it cannot represent detailed local characteristics. This study further explores the problem of spatial non-smoothness based on OLS by adopting the GWR (Table 3) [22,36].

Compared with the OLS models, four GWR models of vibrancy significantly improved the adjusted R square by 3.6% (model 1), 7.7% (model 2), 4.7% (model 3), and 4.7% (model 4). The AICc values of GWR for the four dynamics were lower than those of OLS. Collectively, the local spatial dynamic characteristics of urban vibrancy can be explained better using the GWR model.

As shown by the range of coefficient changes of the independent variables in Table 3, there were noticeable spatial differences in the effects of built environment elements on the vibrancy of the four. The explanatory coefficients of each variable were further visualized and analyzed spatially to characterize the explanatory variables influenced by urban vibrancy in different regions. The results are as follows:

#### 3.3.1. Diversity of Land-Use

**Figure 5:**
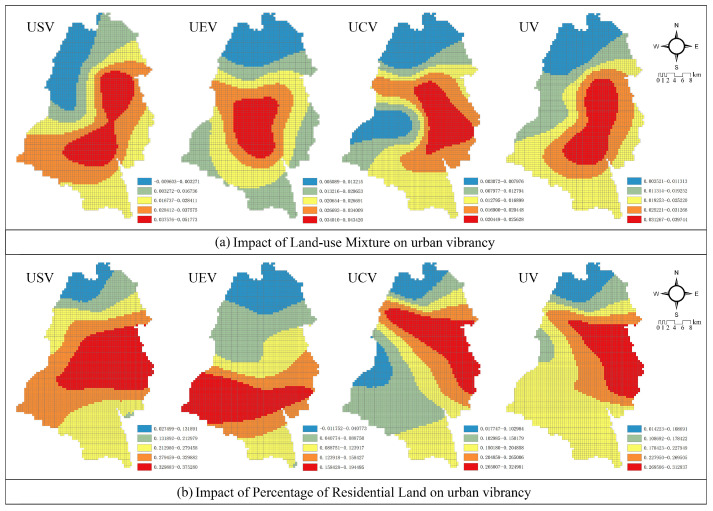
Impact of diversity of land-use on various urban vibrancies. (**a**) Impact of Land-use Mixture on Urban Vibrancy. (**b**) Impact of Percentage of Residential Land on Urban Vibrancy.

*Diversity of Land-use*: As shown in Figure 5a, the Economic Development Zone and Qingchengshan New District form a twin core of the spatial structure. As it can be seen, each type of urban vibrancy benefits from greater land-use variation, however, there is a social vibrancy anomaly in the northwestern part of the city, which is negatively correlated with the degree of land-use mix. It is worth mentioning that the coefficient of land-use mixture fluctuates less (0.003521–0.037944) for comprehensive vibrancy.

*Percentage of Residential Land*: As shown in Figure 5b, increasing the percentage of residential land can effectively promote comprehensive urban vibrancy, particularly in key post-disaster development areas, such as Puyang, Xujia, Tianma, and Chongyi. Furthermore, for economic vibrancy, the spatial distribution of its residential land proportion coefficient is remarkably different from the other three vibrancy, forming a high-value area in Qingchengshan New District.

#### 3.3.2. Supporting Infrastructure

**Figure 6:**
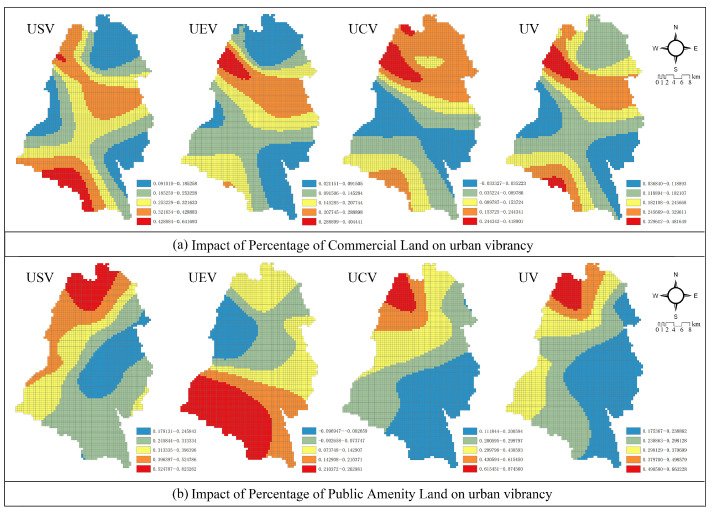
Impact of supporting infrastructure on various urban vibrancies. (**a**) Impact of Percentage of Commercial Land on Urban Vibrancy. (**b**) Impact of Percentage of Public Amenity Land on Urban Vibrancy.

*Percentage of Commercial Land*: Figure 6a shows that the proportion of commercial land is positively correlated with social and economic vibrancy whereas a weak positive correlation or even a negative correlation with cultural vibrancy in the Dujiangyan scenic area on the west side of the city, as well as Chongyi town on the east side. The areas with the greatest positive stimulus to the urban vibrancy level by increasing the proportion of commercial land are mainly concentrated in the vicinity of Puyang Town in the north and Qingcheng Mountain New District in the south, forming two high-value areas, but the positive stimulation gradually diminishes in the central part of the city. In addition, it can be found that increasing the percentage of commercial land-use has the worst stimulating effect on the vibrancy of the central city.

*Percentage of Public Amenity Land*: As shown in Figure 6b, the construction of public amenities has a significant positive correlation with urban vibrancy (particularly social and cultural vibrancy). The spatial pattern reveals that enhancing the construction of public amenities has no significant impact on the stimulation of vibrancy in the old town, but clearly promotes the vibrancy of the distant suburbs of the city (rural areas). For example, in the northern mountainous region, public amenities significantly contribute to social and cultural vibrancy and correlate positively with economic vibrancy at the town-wide level of Mount Qingcheng.

#### 3.3.3. Road Transportation Networks

**Figure 7:**
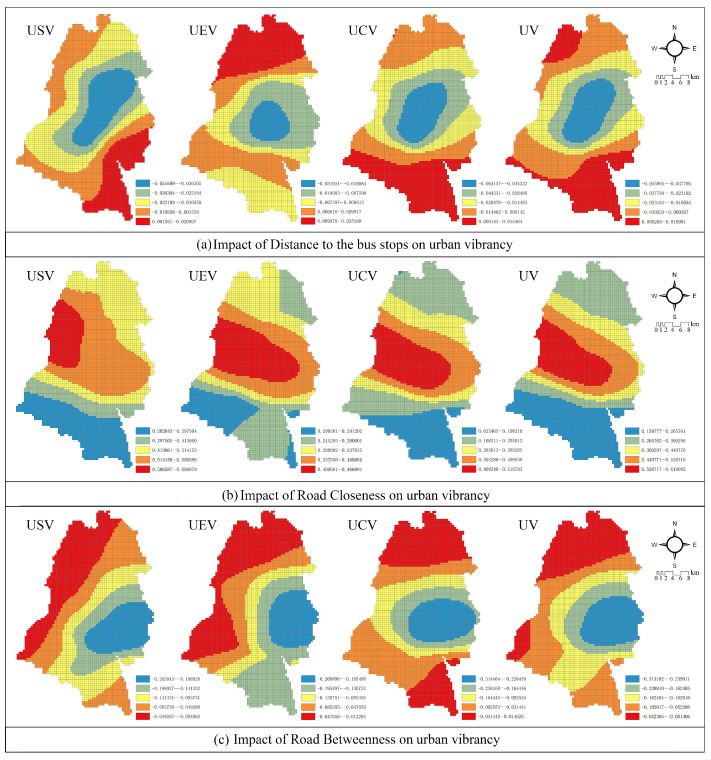
Impact of road transportation networks on various urban vibrancy. (**a**) Impact of Distance to the Bus Stops on Urban Vibrancy. (**b**) Impact of Road Closeness on Urban Vibrancy. (**c**) Impact of Road Betweenness on Urban Vibrancy.

*Distance to the bus stops*: Buses are the main public transportation in Dujiangyan, and the distance to the bus station is usually a key factor affecting people’s choices [6]. According to Figure 7a, in the old town and its neighboring areas, the farther away from the bus station, the lower the convenience for residents to travel and the lower the urban vibrancy. However, in the northernmost and southernmost parts of the city, the distance to the bus stop is positively correlated with the overall vibrancy. By comparing the influence coefficients, it was found that the absolute value of the positive values is too small comparing to the negative values so that even in areas where the explanatory coefficients are positive, the setting of bus stops does not have a significant impact on vibrancy.

*Road Closeness*: As shown in Figure 7b, road closeness is positively correlated with social vibrancy, economic vibrancy, cultural vibrancy, and comprehensive vibrancy. The areas with the most striking effects on urban vibrancy were mainly concentrated in the old town and the Dujiangyan scenic area to the west, and the effect gradually decreases from west to east and from the center to the north and south. Additionally, the vibrancy of Qingchengshan New District, the Puyang Economic Development Zone, and the southern part of the city are less influenced by closeness.

*Road Betweenness*: The betweenness coefficients for all types of vibrancy are negative, which indicates that roads with high betweenness have a suppressive effect on urban vibrancy. As shown in Figure 7c, the significantly inhibited areas were clustered to the east of the downtown area and gradually weakened in all directions. For example, in the downtown area and its eastern part, Xujia, Juyuan, Tianma, and Shongyi towns, urban vibrancy is most strongly inhibited by roads with a high degree of penetration. However, this relationship is reversed in the southernmost town of Liujie and the northernmost town of Longchi in Dujiangyan.

## 4. Discussion

### 4.1. Quantification of Urban Vibrancy and Big Data

Due to its characteristics of large scale, high penetration, and real-time [43,44], urban big data enable researchers to control urban vibrancy more precisely from the perspective of spatial-temporal and social perception, giving conditions for studying human activities and urban spatial relationships [24]. The use of a single source of big data is the dominant approach to quantifying urban vibrancy in current studies; for example, Lan, Gong, Da, and Wen (2020) used NPP-VIIRS Nighttime light data as a proxy for urban vibrancy [45] and Lv, Zheng, and Hu (2022) measured the spatial variation of urban vibrancy in Shenzhen using BHM [46]. However, the one-sidedness of such representations cannot be ignored, while using multi-source big data can provide a more comprehensive understanding of urban space [36]. Most of the current studies in the field of urban vibrancy for quantifying vibrancy using multi-source urban big data have adopted the entropy method for empowerment and integration. For example, Zhu et al. (2021) used this method to integrate both physical and virtual vibrancy [36], Tu et al. (2019) used three vibrancy indicators (Density of POI, density of social media check-ins, and density of mobile phone records) to integrate the comprehensive vibrancy [22]. The method adopted is a single weighting method, which is objective but only considers the information entropy value of each vibrancy, ignoring the correlation and difference between vibrancy [38,39]. The combined evaluation model formed by combining different single assignment methods can better assign [39]. The CRITIC-entropy weighting method used in this study is exactly a portfolio evaluation assignment method. This method fully considers the amount of internal information of each factor and pays attention to their characteristics while retaining the commonality among factors [39]. Additionally, it is currently used in agriculture [47], economics [48], and computer science [49].

With large sample size and high accuracy, multi-source urban data give a multifaceted view of urban vibrancy research. However, everything has two sides. It is also necessary to clearly understand some limitations of big data in application: limited to the popularization of information collection equipment, the acquisition of urban big data is often sampled data, which makes the acquired data deviate from the real situation, and puts forward high requirements for the data supplementation [50]. In addition, the use of multi-source data needs to be combined with the characteristics of the city itself. The same type of data may show different findings in other cities.

### 4.2. City Vibrancy and Influencing Factors

#### 4.2.1. Spatial Distribution Characteristics of Urban Vibrancy

This study quantifies and portrays the economic, social, cultural and comprehensive urban vibrancy of Dujiangyan City by using three sources of big data. The research results can not only reflect the overall vibrancy level of the whole region, but also explain the local characteristics. The distribution of urban vibrancy is characterized by a “double center’’ and a dispersion along the road network (Figure 4). According to the post-disaster reconstruction master plan, in order to solve the resettlement and post-disaster development problems of the disaster victims, the Dujiangyan government adopted the expansion of the original site and the development of new areas to reduce the population pressure in the central city, and the wider suburbs became the key areas for urban expansion and residents’ migration [51]. In addition, from the spatial pattern of social vibrancy, it can be found that the social vibrancy of working days is obviously concentrated in the old town, which shows that despite the planned relocation of residential land after the disaster, the employment concentration in the old city is still high. The social vibrancy of the weekend is obviously concentrated on the expressway, which reflects that the scattered distribution of social resources in Dujiangyan City after reconstruction has increased the needs of residents to move between areas to a certain extent.

#### 4.2.2. Linkage between Built Environment Elements and Urban Vibrancy

(1) Diversity of land-use

According to Yin, Kong, and Zhang (2011), residential land density is a significant indicator of urban development [52]. Although, some scholars have pointed out that a high proportion of residential area will have an inhibitory effect on urban vibrancy [20]. However, according to the findings of this study (Figure 5), increasing the proportion of residential land-use has a significant positive effect on promoting the vibrancy of suburbs with external transportation conditions for urban development. The reason for this may be that the main obstacle to the development of these areas comes from the small size of residential areas, making it difficult to accommodate large populations. In addition, the Qingchengshan New District with tourism as its main industry has a strong demand for short-term tourist accommodation, and the research results also show that increasing the proportion of residential land has a significant effect on promoting its urban vibrancy. Many studies have been conducted to highlight the significant impact of land-use mixing on urban vibrancy [8,18], but in this study, it contributed less to the urban vibrancy of Dujiangyan City than other explanatory variables. One reason may be that the urban reconstruction of Dujiangyan City was based on a clear, chronological plan to unify and optimize the allocation of urban and rural land resources [51]. This makes the allocation of various resources relatively balanced in space.

(2) Supporting infrastructure

Regarding the construction of auxiliary facilities, an increase in the proportion of public amenity land and commercial land can significantly promote the comprehensive urban vibrancy of Dujiangyan City, which is different from some studies in developed cities [20,40]. In this research, the addition of both supporting infrastructures significantly contributes to urban vibrancy quantified by Baidu heat data, nighttime light data, and cultural POI (Table 2), respectively, which Pan et al. (2021) found to have no positive effect on urban vibrancy based on social media check-in data [40].

In terms of commercial land, the influence coefficient of commercial land proportion is positive in the economic development zone, and negative in the west side of the city (Figure 6). It means that the construction of economic development zones can effectively enhance economic and social vibrancy by increasing commercial land and attracting settlement and industry to the city. On the west side of the Giant Panda Habitat Reserve, commercial development will lead to ecological damage, thereby weakening the appeal of cultural tourism and inhibiting cultural vibrancy.

(3) Road transportation networks

Contrary to existing studies that concluded that the prevalence of bus facilities significantly contributes to urban vibrancy [36], it was found that distance to bus stops showed a weak negative correlation with urban vibrancy (Table 2), and the effect of additional bus stops on vibrancy decreases from the central city to the periphery (Figure 7). This reflects that city residents tend to travel in small areas and the central city is still the area with the strongest travel demand.

Regarding the selection of built environment elements, most researchers use road morphology data, such as road network density and the number of intersections, as variables, but rarely explore the relationship with urban vibrancy from the perspective of road network function [20]. This research examines the impact of road closeness on vibrancy and finds that road closeness has the greatest explanatory effect on urban vibrancy in Dujiangyan City. The road closeness index shows a strong positive correlation with overall urban vibrancy, which implies that areas with greater road closeness tend to have greater urban vibrancy.

In terms of local impact (Figure 7), high road closeness enhances the connection between the Dujiangyan scenic area and other regions, which, in turn, promotes tourism development. In addition, there is a greater demand for road networks with high accessibility and centrality in the central city, indicating that it is the dominant occurrence of urban activities. However, roads with a high degree of betweenness significantly inhibit the vibrancy of the central city, as roads with a large amount of traffic flowing through them cause fragmentation of parcels, which adversely affects urban functions and leads to a loss of urban vibrancy.

### 4.3. Recommendations to Promote Urban Vibrancy

The inherent influencing mechanisms of urban vibrancy explored in this study suggest the following recommendations for urban planners: the research results reflect that the population plays a key role in urban vibrancy and is the source of vibrancy. The reconstruction of a post disaster-city is often accompanied by the short-term spatial movement of a large number of people [26]. Therefore, regional resource reconstruction and personnel resettlement planning are critical to promoting the restoration of vibrancy in disaster-stricken areas. According to He et al. (2018), commercial land near residential areas can generally improve the social vibrancy of the area [53], so it is necessary to pay attention to the rational allocation of commercial land in urban planning. In addition, in the process of urban reconstruction and development, especially in the construction of new areas, the spatial layout of public service facilities should be planned and designed according to the habits of local residents, while priority should be given to the central urban area to improve public transport services. In addition, strengthening regional transportation links is crucial to urban development. It is worth mentioning that the impact of road paving on the vibrancy of the area should be considered during planning. The construction of roads with high betweenness in the central area of the city should be avoided as far as possible.

## 5. Conclusions

Many scholars support the idea that urban vibrancy can be defined by the richness of urban activities or the intensity of residents’ activities [10,54,55]. Some scholars also emphasize human activities and interactions as a generation of regional vibrancy [22]. Overall, urban vibrancy can provide a visual reflection of urban development, and exploring the causes of its generation can promote healthy urban development.

Most recent studies have focused on cities or regions with steady development as the study areas [8,41], and rarely evaluated cities that have been rebuilt after suffering from large disasters. Dujiangyan, which was heavily hit by the Wenchuan earthquake, is now in the revitalization and development stages of reconstruction planning. This study uses the CRITIC-entropy method to objectively assign three vibrancy indicators (USV, UEV, and UCV), and then quantifies the comprehensive urban vibrancy to characterize the current status of post-disaster recovery. The OLS and GWR models were used to investigate the correlation between built environment elements and urban vibrancy, conduct a comprehensive analysis and evaluation of post-disaster urban development in Dujiangyan City, and discuss planning initiatives in the built environment to stimulate urban vibrancy. The main findings of the study are as follows: (1) urban vibrancy indicators obtained from single-source data have limitations in depicting urban vibrancy; however, the comprehensive urban vibrancy model based on the CRITIC-entropy method used in this study is an all-encompassing characterization of urban vibrancy. (2) The urban vibrancy of Dujiangyan City, which was restored and rebuilt after the disaster, shows the geospatial characteristics of a bi-central structure and high continuity. The distribution of urban vibrancy in the old town shows high spatial continuity, whereas the distribution in the New Districts shows spatial aggregation. Urban road networks play a connecting role in regional vibrancy. (3) The built environment elements had a similar global impact on different urban vibrancy trends. The closeness of roads, land-use mixture, proportion of residential land, commercial land, and public amenity land demonstrate a positive correlation with the level of urban vibrancy, while road betweenness and distance to bus stops demonstrate a negative correlation with urban vibrancy. (4) The effects of public amenity construction, road closeness, and the allocation of land-use attributes on urban vibrancy demonstrate significant spatial heterogeneity.

This study can be further developed in the following ways. First, owing to the difficulty of data acquisition, this study did not explore the change in vibrancy from a temporal perspective; therefore, geographically and temporally weighted regression (GTWR) will be introduced in the future to investigate the spatio-temporal changes of vibrancy. Additionally, in terms of spatial accuracy, the unit scale of the study is large because of the limitation of the resolution of nighttime light data, whereas the nighttime light data produced by the China Luojia-1 satellite with a higher spatial resolution will be used in further research to observe the finer spatial distribution of urban vibrancy. Finally, this study only evaluated Dujiangyan city, and, the framework will be implemented in other affected cities in the future to improve the vibrancy evaluation system for post-disaster reconstruction.

## Figures and Tables

**Table 1:** Descriptive statistics of variables.

Index	Abbreviation	Mean	Standard Deviation
Land-use Mixture Index	LMI	0.17615635	0.31882732
Residential Land Index	RLI	0.03536129	0.13483714
Commercial Land Index	CLI	0.01238231	0.06834059
Public Amenity Land Index	PALI	0.00598902	0.05220615
Distance Index	DI	0.21211727	0.30846335
Road Closeness Index	RCI	0.08632102	0.15405602
Road Betweenness Index	RBI	0.06313485	0.09376886

**Table 2:** The results for ordinary least squares regression.

OLS Models	Coef	Beta Coef	*p*-Value	Std.	Vif	R Square	Adjusted R Square	AICc
Model 1 (USV) ^1^						0.721	0.720	−10,023.679
Intercept	0.001		0.339	0.005				
RLI	0.343	0.335	0.000	0.074	1.661			
CLI	0.291	0.144	0.000	0.097	1.163			
PALI	0.280	0.106	0.000	0.113	1.079			
DI	−0.030	−0.067	0.000	0.017	1.142			
RCI	0.490	0.547	0.000	0.131	2.966			
RBI	−0.163	−0.111	0.000	0.071	1.696			
Model 2 (UCV) ^1^						0.668	0.668	−13,120.566
Intercept	0.001		0.440	0.004				
LMI	0.014	0.050	0.000	0.006	1.357			
RLI	0.224	0.345	0.000	0.072	1.661			
CLI	0.082	0.064	0.000	0.084	1.163			
PALI	0.203	0.121	0.000	0.148	1.079			
DI	−0.034	−0.121	0.000	0.020	1.142			
RCI	0.325	0.573	0.000	0.142	2.966			
RBI	−0.164	−0.175	0.000	0.097	1.696			
Model 3 (UEV) ^1^						0.695	0.695	−13,626.702
Intercept	0.011		0.000	0.004				
LMI	0.024	0.087	0.000	0.009	1.357			
RLI	0.144	0.226	0.000	0.056	1.661			
CLI	0.173	0.137	0.000	0.072	1.163			
PALI	0.162	0.098	0.000	0.096	1.079			
DI	−0.013	−0.046	0.000	0.012	1.142			
RCI	0.351	0.629	0.000	0.074	2.966			
RBI	−0.145	−0.158	0.000	0.073	1.696			
Model 4 (UV) ^1^						0.777	0.776	−12,905.897
Intercept	0.004		0.000	0.003				
LMI	0.023	0.066	0.000	0.009	1.357			
RLI	0.268	0.330	0.000	0.060	1.661			
CLI	0.192	0.120	0.000	0.080	1.163			
PALI	0.244	0.116	0.000	0.092	1.079			
DI	−0.031	−0.087	0.000	0.017	1.142			
RCI	0.435	0.612	0.000	0.131	2.966			
RBI	−0.181	−0.155	0.000	0.087	1.696			

^1^ By changing only the search radius (400 m, 800 m, 1200 m, 5000 m, 8000 m, Nm) of road closeness and betweenness and repeating the OLS experiment, The research find all built environment elements have the best interpretation of USV, UEV, UCV, and UV when the search radius is 5000 m (refer to Table A3, Table A4, Table A5 and Table A6 in Appendix A). Therefore, the road closeness and betweenness with the search radius of 5000 m are selected.

**Table 3:** The results for geographically weighted regression.

GWR Models	Min	Median	Max	Mean	R Square	Adjusted R Square	AICc
Model 1 (USV)					0.758	0.756	−10,578.473
Intercept	−0.021	0.000	0.007	−0.001			
LMI	−0.010	0.028	0.052	0.024			
RLI	0.027	0.292	0.375	0.278			
CLI	0.091	0.262	0.642	0.267			
PALI	0.179	0.293	0.823	0.333			
DI	−0.052	−0.014	0.021	−0.016			
RCI	0.203	0.498	0.686	0.455			
RBI	−0.264	−0.093	−0.004	−0.106			
Model 2 (UCV)					0.748	0.745	−14,210.057
Intercept	−0.018	0.000	0.005	−0.001			
LMI	0.003	0.015	0.026	0.014			
RLI	0.018	0.166	0.325	0.180			
CLI	−0.033	0.098	0.419	0.110			
PALI	0.112	0.238	0.875	0.281			
DI	−0.063	−0.015	0.015	−0.017			
RCI	0.025	0.226	0.517	0.250			
RBI	−0.314	−0.069	0.015	−0.102			
Model 3 (UEV)					0.744	0.741	−14,307.063
Intercept	0.007	0.011	0.026	0.012			
LMI	0.005	0.022	0.043	0.022			
RLI	−0.012	0.110	0.194	0.107			
CLI	0.021	0.131	0.404	0.142			
PALI	−0.097	0.111	0.263	0.118			
DI	−0.032	0.000	0.025	−0.001			
RCI	0.208	0.304	0.467	0.323			
RBI	−0.267	−0.086	−0.012	−0.104			
Model 4 (UV)					0.825	0.823	−13,864.880
Intercept	−0.009	0.003	0.011	0.003			
LMI	0.004	0.023	0.040	0.022			
RLI	0.014	0.216	0.313	0.213			
CLI	0.037	0.171	0.482	0.188			
PALI	0.175	0.260	0.663	0.285			
DI	−0.056	−0.010	0.011	−0.014			
RCI	0.160	0.367	0.619	0.377			
RBI	−0.313	−0.097	−0.001	−0.119			

## Data Availability

Not applicable.

## Abbreviations

The following abbreviations are used in this manuscript:

CRITIC	Criteria Importance Though Inter-criteria Correlation
BHM	Baidu Heat Map
NTL	Nighttime light
POI	Point-of-interest
Suomi-NPP	The Suomi National Polar-orbiting Partnership
VIIRS	The visible infrared imaging radiometer suit
GDP	Gross domestic product
OLS	Ordinary least squares
GWR	Geographically weighted regression
GTWR	Geographically and temporally weighted regression
USV	Urban Social Vibrancy
UEV	Urban Economic Vibrancy
UCV	Urban Cultural Vibrancy
UV	Urban Comprehensive Vibrancy
LMI	Land-use Mixture Index
RLI	Residential Land Index
CLI	Commercial Land Index
PALI	Public Amenity Land Index
DI	Distance Index
RCI	Road Closeness Index
RBI	Road Betweenness Index

## Appendix A

**Table A1:** POI data classification.

Integration Category	Specific Type	Counts
Traffic service	Bus stop/Place name and address information	2187
Education and culture	Education and culture services/Museums/Libraries/Theatres and concert halls/ Exhibition halls/Scientific research institutions and schools/Place name and address information	1095
Catering service	Catering services/Place name and address information	5841
Shopping service	Shopping services/Cars or motorcycle sales/Place name and address information	10,982
Life service	Life services/Public facilities/Access facilities/Indoor facilities/Place name and address information	6882
Corporate business	Access facilities/Place name and address information	2190
Government body	Government agencies/Social organizations/Place name and address information	1912
Accommodation service	Accommodation services/Place name and address information	1928
Residential area	Business residence/Place name and address information	1878
Health care	Health care services/Place name and address information	1502
Leisure and entertainment	Sports and leisure services/Famous tourist sites/Place name and address information	1772
Financial service	Financial insurance services/Place name and address information	431

**Table A2:** The built environment elements selected for this study suggest that the determinants of urban vibrancy.

Variables	Definition	Mean	Standard Deviation	Data Source
Land-use Mixture	the Simpson index of the mixed use of 12 POI types	0.157	0.285	(b) ^1^
Percentage of Residential Land	The proportion of residential land areas in each unit	0.031	0.119	(a) ^1^
Percentage of Commercial Land	The proportion of commercial land areas in each unit	0.011	0.063	(a) ^1^
Percentage of Public Amenity Land	The proportion of public amenity land areas in each unit	0.004	0.035	(a) ^1^
Road Closeness	a measure of how easy it is for a road to reach other roads within the search radius	34.370	61.333	(c) ^1^
Road Betweenness	a measure of the probability of a road segment being passed by traffic flow	770.402	1144.076	(c) ^1^
Distance to the bus stops	minimum distance from the grid center point to the nearest bus stop	180.155	261.953	(d) ^1^

^1^ (a) land-use data; (b) point-of-interest (POI) dataset; (c) road networks; (d) public transportation stations.

**Table A3:** The resulting for ols model between USV and built environment variables under different search radius.

OLS Models	Coef	Beta Coef	*p*-Value	R Square	Ajusted R Square
Model 1 (r = 400)				0.629860839	0.629240989
Intercept	0.000		0.842		
LMI	0.057	0.132	0.000		
RLI	0.541	0.528	0.000		
CLI	0.437	0.216	0.000		
PALI	0.385	0.145	0.000		
DI	−0.007	−0.017	0.098		
RCI	0.171	0.199	0.000		
RBI	−0.081	−0.085	0.000		
Model 2 (r = 800)				0.682883193	0.682352136
Intercept	−0.004		0.025		
LMI	0.041	0.094	0.000		
RLI	0.432	0.422	0.000		
CLI	0.362	0.179	0.000		
PALI	0.336	0.127	0.000		
DI	−0.022	−0.049	0.000		
RCI	0.362	0.387	0.000		
RBI	−0.112	−0.118	0.000		
Model 3 (r = 1200)				0.716157379	0.715682045
Intercept	−0.004		0.024		
LMI	0.032	0.075	0.000		
RLI	0.361	0.353	0.000		
CLI	0.326	0.162	0.000		
PALI	0.294	0.111	0.000		
DI	−0.022	−0.050	0.000		
RCI	0.503	0.474	0.000		
RBI	−0.078	−0.084	0.000		
Model 4 (r = 5000)				0.720615665	0.720147797
Intercept	0.001		0.339		
LMI	0.025	0.057	0.000		
RLI	0.343	0.335	0.000		
CLI	0.291	0.144	0.000		
PALI	0.280	0.106	0.000		
DI	−0.030	−0.067	0.000		
RCI	0.490	0.547	0.000		
RBI	−0.163	−0.111	0.000		
Model 5 (r = 8000)				0.683390959	0.682860752
Intercept	0.000		0.825		
LMI	0.033	0.075	0.000		
RLI	0.414	0.405	0.000		
CLI	0.329	0.163	0.000		
PALI	0.321	0.121	0.000		
DI	−0.033	−0.075	0.000		
RCI	0.302	0.432	0.000		
RBI	−0.119	−0.078	0.000		
Model 6 (r = N)				0.64689031	0.646298978
Intercept	−0.005		0.002		
LMI	0.045	0.103	0.000		
RLI	0.498	0.486	0.000		
CLI	0.402	0.199	0.000		
PALI	0.359	0.136	0.000		
DI	−0.027	−0.061	0.000		
RCI	0.126	0.256	0.000		
RBI	0.023	0.010	0.323		

**Table A4:** The resulting for ols model between UCV and built environment variables under different search radius.

OLS Models (UCV)	Coef	Beta Coef	*p*-Value	R Square	Ajusted R Square
Model 1 (r = 400)				0.558	0.557
Intercept	0.000				
LMI	0.038	0.137	0.000		
RLI	0.361	0.556	0.000		
CLI	0.190	0.148	0.000		
PALI	0.270	0.161	0.000		
DI	−0.014	−0.048	0.000		
RCI	0.044	0.080	0.000		
RBI	−0.030	−0.049	0.000		
Model 2 (r = 800)				0.601	0.600
Intercept	−0.002		0.057		
LMI	0.027	0.097	0.000		
RLI	0.301	0.464	0.000		
CLI	0.145	0.113	0.000		
PALI	0.245	0.146	0.000		
DI	−0.026	−0.093	0.000		
RCI	0.182	0.306	0.000		
RBI	−0.070	−0.117	0.000		
Model 3 (r = 1200)				0.658	0.658
Intercept	−0.003		0.010		
LMI	0.018	0.065	0.000		
RLI	0.241	0.371	0.000		
CLI	0.110	0.085	0.000		
PALI	0.212	0.126	0.000		
DI	−0.031	−0.109	0.000		
RCI	0.314	0.467	0.000		
RBI	−0.068	−0.115	0.000		
Model 4 (r = 5000)				0.668	0.668
Intercept	0.001		0.440		
LMI	0.014	0.050	0.000		
RLI	0.224	0.345	0.000		
CLI	0.082	0.064	0.000		
PALI	0.203	0.121	0.000		
DI	−0.034	−0.121	0.000		
RCI	0.325	0.573	0.000		
RBI	−0.164	−0.175	0.000		
Model 5 (r = 8000)				0.610	0.609
Intercept	−0.001		0.540		
LMI	0.021	0.078	0.000		
RLI	0.284	0.437	0.000		
CLI	0.119	0.093	0.000		
PALI	0.237	0.141	0.000		
DI	−0.033	−0.117	0.000		
RCI	0.174	0.391	0.000		
RBI	−0.113	−0.118	0.000		
Model 6 (r = N)				0.569	0.568
Intercept	−0.003		0.024		
LMI	0.031	0.113	0.000		
RLI	0.341	0.526	0.000		
CLI	0.172	0.134	0.000		
PALI	0.260	0.155	0.000		
DI	−0.025	−0.087	0.000		
RCI	0.049	0.156	0.000		
RBI	0.007	0.005	0.679		

**Table A5:** The resulting for ols model between UEV and built environment variables under different search radius.

OLS Models(UEV)	Coef	Beta Coef	*p*-Value	R Square	Ajusted R Square
Model 1 (r = 400)				0.567	0.567
Intercept	0.010		0.000		
LMI	0.048	0.177	0.000		
RLI	0.288	0.452	0.000		
CLI	0.282	0.224	0.000		
PALI	0.236	0.143	0.000		
DI	0.006	0.022	0.048		
RCI	0.089	0.167	0.000		
RBI	−0.050	−0.085	0.000		
Model 2 (r = 800)				0.608	0.608
Intercept	0.008		0.000		
LMI	0.039	0.144	0.000		
RLI	0.229	0.360	0.000		
CLI	0.242	0.192	0.000		
PALI	0.209	0.127	0.000		
DI	−0.002	−0.008	0.457		
RCI	0.196	0.336	0.000		
RBI	−0.069	−0.117	0.000		
Model 3 (r = 1200)				0.653	0.652
Intercept	0.008		0.000		
LMI	0.032	0.119	0.000		
RLI	0.178	0.280	0.000		
CLI	0.213	0.170	0.000		
PALI	0.181	0.110	0.000		
DI	−0.004	−0.016	0.091		
RCI	0.304	0.460	0.000		
RBI	−0.059	−0.101	0.000		
Model 4 (r = 5000)				0.695	0.695
Intercept	0.011		0.000		
LMI	0.024	0.087	0.000		
RLI	0.144	0.226	0.000		
CLI	0.173	0.137	0.000		
PALI	0.162	0.098	0.000		
DI	−0.013	−0.046	0.000		
RCI	0.351	0.629	0.000		
RBI	−0.145	−0.158	0.000		
Model 5 (r = 8000)				0.655	0.655
Intercept	0.009		0.000		
LMI	0.028	0.105	0.000		
RLI	0.190	0.298	0.000		
CLI	0.194	0.155	0.000		
PALI	0.191	0.116	0.000		
DI	−0.016	−0.058	0.000		
RCI	0.230	0.527	0.000		
RBI	−0.140	−0.148	0.000		
Model 6 (r = N)				0.589	0.588
Intercept	0.006		0.000		
LMI	0.039	0.145	0.000		
RLI	0.260	0.408	0.000		
CLI	0.259	0.206	0.000		
PALI	0.220	0.134	0.000		
DI	−0.008	−0.029	0.010		
RCI	0.075	0.246	0.000		
RBI	0.018	0.013	0.230		

**Table A6:** The resulting for ols model between UV and built environment variables under different search radius.

OLS Models	Coef	Beta Coef	*p*-Value	R Square	Ajusted R Square
Model 1 (r = 400)				0.655	0.654
Intercept	0.003		0.013		
LMI	0.053	0.154	0.000		
RLI	0.448	0.551	0.000		
CLI	0.328	0.205	0.000		
PALI	0.335	0.160	0.000		
DI	−0.007	−0.020	0.040		
RCI	0.107	0.156	0.000		
RBI	−0.057	−0.076	0.000		
Model 2 (r = 800)				0.708	0.708
Intercept	0.000		0.761		
LMI	0.039	0.114	0.000		
RLI	0.364	0.448	0.000		
CLI	0.269	0.168	0.000		
PALI	0.298	0.142	0.000		
DI	−0.021	−0.058	0.000		
RCI	0.271	0.365	0.000		
RBI	−0.093	−0.124	0.000		
Model 3 (r = 1200)				0.760	0.759
Intercept	9.172 0.000		0.937		
LMI	0.030	0.087	0.000		
RLI	0.294	0.362	0.000		
CLI	0.230	0.144	0.000		
PALI	0.259	0.124	0.000		
DI	−0.024	−0.067	0.000		
RCI	0.418	0.497	0.000		
RBI	−0.078	−0.106	0.000		
Model 4 (r = 5000)				0.777	0.777
Intercept	0.004		0.000		
LMI	0.023	0.066	0.000		
RLI	0.268	0.330	0.000		
CLI	0.192	0.120	0.000		
PALI	0.244	0.116	0.000		
DI	−0.031	−0.087	0.000		
RCI	0.435	0.612	0.000		
RBI	−0.181	−0.155	0.000		
Model 5 (r = 8000)				0.725	0.724
Intercept	0.003		0.023		
LMI	0.030	0.089	0.000		
RLI	0.336	0.414	0.000		
CLI	0.229	0.143	0.000		
PALI	0.283	0.135	0.000		
DI	−0.032	−0.091	0.000		
RCI	0.260	0.467	0.000		
RBI	−0.141	−0.117	0.000		
Model 6 (r = N)				0.673	0.673
Intercept	−0.001		0.455		
LMI	0.043	0.125	0.000		
RLI	0.415	0.511	0.000		
CLI	0.301	0.188	0.000		
PALI	0.317	0.151	0.000		
DI	−0.024	−0.066	0.000		
RCI	0.090	0.230	0.000		
RBI	0.017	0.009	0.337		
